# The dilemma of the split between theory and reality as experienced by primary healthcare professionals: a mixed methods study of evidence-based practice in a primary care context

**DOI:** 10.1186/s12875-023-02237-9

**Published:** 2024-01-05

**Authors:** Tobias Abelsson, Ann-Kristin Karlsson, Helena Morténius, Amir Baigi, Stefan Bergman

**Affiliations:** 1 Primary Healthcare Unit, Department of Public Health and Community Medicine, Institute of Medicine, The Sahlgrenska Academy, University of Gothenburg, Gothenburg, Sweden; 2 Department of Child and Adolescent Mental Health, Child and Adolescent Psychiatry, Region Halland, Halmstad, Sweden; 3 Department of Research and Development, Region Halland, Halmstad, Sweden; 4 Spenshult Research and Development Centre, Halmstad, Sweden; 5 Primary Healthcare Centre Bäckagård, Region Halland, Halmstad, Sweden

**Keywords:** EBM, EBP, Healthcare professionals, Mixed method, Experience, Perception

## Abstract

**Background:**

Primary care depends upon a good information flow across professional and structural boundaries to provide the best care for patients. Previous research has mainly focused on Evidence-Based Practice (EBP) within specific professions. Mapping of pan-professional experiences of and attitudes to EBP in publicly funded clinical practice is necessary to deepen the understanding of EBP and its implementation. Thus, this study aimed to investigate healthcare professionals’ experiences of and attitudes towards working in accordance with EBP in primary care.

**Methods:**

The study used a convergent mixed methods design divided into two strands: a quantitative enquiry tool (Evidence-Based Practice Attitude Scale, EBPAS) and a set of qualitative interviews analysed by means of qualitative content analysis. Both strands included all primary care employees with patient interaction in the studied county (n = 625), including doctors, nurses, physiotherapists, psychologists and assistant nurses. Out of the original 625 healthcare professionals, 191 finished the first strand and 8 volunteered for the second strand (2 nurses, 2 physiotherapists, 1 psychiatrist and 3 doctors).

**Results:**

The EBPAS value of 2.8 (max 4) indicated a generally positive attitude towards EBP amongst the population, which was also evident in the interviews. However, there were additional experiences of not having the ability or resources to engage in EBP. This was illustrated by the theme that emerged from the qualitative content analysis: “The dilemma of the split between theory and reality”. Due to the organisational and managerial focus on efficiency rather than quality of care, there were few or no incentives for promoting individual educational or research development.

**Conclusions:**

Although the general attitude towards EBP is positive, experiences of practising it differ. There is a need to increase knowledge of EBP concepts, requirements and implementation in the clinical setting. The absence of opportunities to do research and collegial debate about new ways of finding and implementing research-based evidence results might influence the quality of care.

**Supplementary Information:**

The online version contains supplementary material available at 10.1186/s12875-023-02237-9.

## Background

Evidence-Based practice (EBP) is defined as merging patients’ values and preferences with clinical expertise and best available evidence to provide the highest quality care possible to patients [1, 2]. EBP is recognised as a core skill by the World Health Organisation and healthcare professionals (HPs) in primary care (PC) [3–5]. The need for a common understanding of EBP and its application was identified about two decades ago [6]. Nevertheless, the need for a common, non-profession-specific definition of EBP and the skills required to practise it still seems to exist [1, 3, 4, 7–13]. Complete understanding and practice of the EBP process are dependent on a range of factors from individual to organisational [6]. Of these factors, information management skills such as critical thinking and analysis of complex information are vital [3, 4, 6, 8, 11].

The PC mission is to be the first line of healthcare provision for treating every type of illness in all segments of the population [7, 14, 15]. To handle this huge mission and deliver the best available care, the principles of EBP are adhered to [7, 8].

The research of attitudes towards EBP and its practical use has so far generally focused on three major areas: (1) Case studies and meta-analyses covering specific stakeholder groups, such as patients in need of information during certain therapies [16–18]. (2) Information dissemination and use of EBP within and between clinical professions [4, 5, 19–21]. (3) EBP usage on strategic levels within organisations [8, 22, 23].

Few articles addressing nursing staff members’ attitudes towards EBP and their willingness to engage in research activities have been found [24, 25]. However, some systematic reviews on the attitudes and competencies related to EBP practice and implementation have been identified [3, 4]. These conclude that EBP can enhance healthcare safety and patient outcomes as well as increase economic gain [3, 4, 25].

Despite earlier research, barriers to achieving a fully integrated healthcare organisation that implements EBP in its truest sense still exist [3, 20, 25, 26]. The results of these studies identified lack of time and resources on all levels as the largest barriers that prevent HPs from fully practising EBP [7, 8, 25, 27–29]. Such barriers ultimately resulted in ethical stress amongst managers [16], whose attitudes towards EBP have also been proven to influence the local organisational implementation of EBP [3, 4, 7, 8]. Besides organisational and managerial influence, the individual attitudes of HPs have been identified as an important factor in EBP practice and implementation [3, 20, 25, 30]. Their attitudes have been investigated in previous studies employing the verified Evidence-Based Practice Attitude Scale (EBPAS) [26, 31, 32].

The above leads to the conclusion that there seems to be a general knowledge gap concerning the attitudes of all HP categories towards use of the new research-based methods described by the EBP guidelines/protocol. Thus, the aim of this study was to investigate HPs’ attitudes towards and experiences of working according to EBP in PC. To obtain a more comprehensive result, the present study will apply a mixed methods design involving both qualitative and quantitative methodologies.

## Methods

### Design and setting

The study used a convergent mixed methods design as described by Creswell [33]. Two study strands, a quantitative and a qualitative, were merged into a single result [33]. The first strand was based on the responses to a validated EBPAS instrument about attitudes towards EBP completed by PC HPs in a Swedish healthcare region [31]. The second consisted of a qualitative content analysis of interviews with different professional categories within PC [34].

### The quantitative strand

#### Recruitment and study population

The quantitative data collection took place online with the aim of recruiting HPs in all public PC centres in the chosen healthcare region, in total 625 individuals. Inclusion criteria were HPs with experience of patient contact. The exclusion criterion was being employed in a managerial position.

The addresses of all eligible HPs were received from the central PC management in the studied region. An e-mail with a link to the survey was sent to all eligible HPs. One week after the initial invitation, a reminder was sent to those who had not yet completed the survey. This process was repeated on two further occasions, so that each HP who had not responded received a maximum of four invitations to take part in the survey.

#### Questionnaire design and construction

The questionnaire used was the validated EBPAS instrument (Appendix 1), previously shown to have high overall reliability [32]. The instrument was created and validated in English, after which it was translated into Swedish. Although originally constructed for use in a psychiatric staff context, the instrument was considered appropriate for all personnel who are obliged to work in accordance with EBP. EBPAS encircles four domains of psychometric measurement to describe attitudes towards adopting EBP [31]. This is done in four dimensions; Required to work according to EBP (Requirements), Intuitive appeal (Appeal), Openness to new practice (Openness) and finally perceived divergence (Divergence) from established EBP-practice [31]. In addition to the instrument, five questions of a demographic nature were added; age, sex, education, number of years employed and profession.

Data were normalised and prepared for use according to the EBPAS instructions for items and scoring developed by Aarons [31, 32]. More specifically, this meant that each item was ordered on a numeric scale from 0 to 4 (0 = not at all, 1 = to a slight extent, 2 = to a moderate extent, 3 = to a great extent, 4 = to a very great extent) [31]. Each of the 15 items in the questionnaire was then grouped into four subscales labelled: Requirements, Appeal, Openness and Divergence, the content of which is illustrated in Table 1. A change in the scoring was made for the divergence subscale, where all items were scored in reverse when calculating the total mean score for the domain [31].

**Table 1:** Means of the EBPAS subdomains

	Mean (max = 4)	SD
*Requirements*	2.8	0.9
*Appeal*	3.1	0.6
*Openness*	2.9	0.6
*Divergence*	1.5	0.6
*Total*	2.8	0.5

#### Statistical analyses

The statistical analyses were performed using IBM SPSS Version 28.0. Descriptive data were presented with percentages, means and standard deviations (SD). Main dependent variables were the EBPAS total and subdomain scores. Independent explanatory variables were age, sex, experience, education and occupation. Differences in mean values for each independent variable were analysed with Student´s t-test. Significance level was set to *p* < 0.05.

### The qualitative strand

#### Study procedure

The same HPs who received the EBPAS instrument were invited to participate in the qualitative strand, which comprised six individual interviews via telephone or video call and one interview carried out face to face with two participants, giving a total of eight participants. The participants recruited for the qualitative strand represented most of the major professional groups (doctors, psychologists, physiotherapists and nurses).

#### Study setting and data collection

An invitation was sent by e-mail to the same recipients as in the quantitative strand, inviting them to attend the interviews. A reminder was sent after one week. All but one interview, which was conducted face to face with two participants, were performed via telephone or video. The interviews, the duration of which was 30 to 50 min, were performed by the first author using open-ended questions, the responses to which were deepened by follow-up questions. The interviews began with an introduction covering the background and aim of the study. An interview guide (Appendix 2) was used to collect information on themes from the EBPAS questionnaire. The resulting data were then transcribed verbatim by the first author.

#### Qualitative data analysis

The data in the qualitative strand **were** analysed using the qualitative content analysis method presented by Graneheim and Lundman in 2004 [34] with additional insights from Lindgren, Lundgren and Graneheim in 2020, such as the division of the process into five steps [35]:

##### De-contextualisation phase

1. Immersion of manifest data: All available verbatim transcripts were read through until a full understanding of the text was achieved. This was done by four of the co-authors in the research group.
2. Meaning units relevant to the aim of the study were identified by the four co-authors and discussed until consensus was achieved.
3. Meaning units were condensed and coded. This was done by the first author. Their relevance was discussed within the group until consensus was achieved.

##### Re-contextualisation phase

4. The codes were then grouped into subcategories and categories in accordance with the similarities and differences in their content. The authors discussed the subcategories and categories until agreement was reached.
5. Abstraction of the latent content: the latent meaning contained in the resulting categories was discussed amongst the authors resulting in an overarching theme.

The goal of the parallel qualitative analyses conducted by the co-authors during the analysis process was to enhance reliability and ensure quality.

### The convergent mixed methods analysis

The purpose of the convergent design was to merge data from the two different strands into a comprehensive result, in order to identify complementary aspects of the data and increase the understanding of EBP in PC. The convergent analysis was performed in four steps in accordance with Creswell’s description of the process [36].

The first two steps involved collecting and analysing the qualitative and quantitative data separately in accordance with each of the chosen methods. The third step, “Interface”, consisted of creating a joint table that illustrated the quantitative EBPAS subdomains within the categories created in the qualitative analysis. In the fourth step, the results from the EBPAS, total score and occurrence of individual subdomains in the quantitative results were merged with the qualitative results and discussed as a unit.

## Results

### The quantitative strand

Of the 190 participants, 79.5% were female and 20.5% male. Most were within the 31 to 40-year age cohort and had a master’s degree (Table 2). The means for the EBPAS total and subdomain scores were calculated for the whole dataset (Table 1). Differences in mean values for each independent variable are presented in Table 1. There were four subdomains: Requirement, Appeal, Openness and Divergence (overall Cronbach’s alpha = 0.76).

**Table 2:** Descriptive statistics of the background variables. N = 190

		*N*	*%*
**Sex**	*Male*	39	20.5
	*Female*	151	79.5
**Age**	*21–30*	21	11.1
	*31–40*	53	27.9
	*41–50*	44	23.2
	*51–60*	47	24.7
	*61-*	25	13.2
**Experience**	*-5*	52	27.4
	*6–10*	36	18.9
	*11–15*	31	16.3
	*16–19*	16	8.4
	*20-*	55	28.9
**Education**	*High school*	19	10
	*Candidate*	69	36.3
	*Master*	74	38.6
	*PhD*	3	1.6
	*Other*	25	13.2
**Occupation**	*Occupational therapist*	13	6.8
	*Assistant nurse*	23	12.1
	*Registered nurse*	66	34.7
	*Medical specialist*	21	11.1
	*Psychologist*	15	7.9
	*Physiotherapist*	27	14.2
	*Intern and resident*	17	8.9
	*Medical social worker*	7	3.7
	*Rehabilitation ass.*	1	0.5

**Table 3:** Independent demographic variables related to the EBPAS subdomains. Statistically significant differences in mean values are indicated by bold italics.Low education = high school and lower, high education = candidate graduates and higher

		*Requirements*				*Appeal*			*Openness*			*Divergence*			*Total*		
		N	Mean	SD	*P*	Mean	SD	*P*	Mean	SD	*P*	Mean	SD	*P*	Mean	SD	*P*
*Age*	**> 40**	116	2.8	0.8	0.656	3.1	0.6	0.083	2.8	0.6	0.334	***1.6***	***0.6***	***0.010***	***2.8***	***0.5***	***0.040***
	**≤ 40**	74	2.8	0.9		3.2	0.6		2.9	0.6		***1.4***	***0.6***		***2.9***	***0.4***	
*Sex*	**M**	***39***	***2.4***	***0.8***	***0.004***	3.0	0.6	0.121	2.7	0.8	0.097	1.4	0.6	0.136	2.7	0.5	0.071
	**F**	***151***	***2.9***	***0.9***		3.2	0.6		2.9	0.6		1.6	0.6		2.9	0.4	
*Experience*	**> 10**	***102***	***2.9***	***0.8***	***0.023***	3.1	0.6	0.333	2.9	0.6	0.879	***1.7***	***0.6***	***0.020***	2.8	0.5	0.614
	**≤ 10**	***88***	***2.6***	***0.9***		3.2	0.6		2.9	0.6		***1.4***	***0.6***		2.8	0.5	
*Education*	**High**	171	2.8	0.8	0.159	3.2	0.6	0.135	2.9	0.6	0.760	***1.9***	***0.6***	***0.005***	2.8	0.5	0.710
	**Low**	19	3.1	1.0		2.9	0.8		2.6	0.6		***1.5***	***0.6***		2.6	0.5	

In the subdomain Requirement, significant differences were found between sex and length of professional experience. Females scored higher than males, as did those with over ten years of professional experience (Table 3). In the subdomains Appeal and Openness, no statistically significant differences in mean values were observed for any of the independent variables. In the Divergence subdomain, there were significant differences between age, length of professional experience and educational level. Participants older than 40 years had a higher score than younger participants, indicating greater divergence regarding EBP, which was also the case for those with longer professional experience and low educational level (High school level) (Table 3). In terms of the total score, there were significant differences in age and sex, with females 40 years or under having higher scores (Table 3).

Mean values for the four subdomains and total scores concerning occupation are presented in Table 4. Of particular interest is the comparison of specialist doctors, interns and residents, as they have the same educational background but different lengths of professional experience. Statistically significant differences were only seen for the subdomain Requirements (*p* = 0.004) and for the total score (*p* = 0.047), where interns and residents scored higher than specialists.

**Table 4:** Professional category and mean variables related to the EBPAS subdomains

	Requirements			Appeal		Openness		Divergence		Total	
	*N*	*Mean*	*SD*	*Mean*	*SD*	*Mean*	*SD*	*Mean*	*SD*	*Mean*	*SD*
*Occupational therapist*	13	3.4	0.6	3.4	0.5	3.1	0.5	1.3	0.5	3.1	0.4
*Assistant nurse*	23	3.1	0.9	2.9	0.7	2.9	0.5	1.8	0.6	2.7	0.5
*Registered nurse*	66	2.9	0.8	3.1	0.6	2.9	0.6	1.6	0.6	2.8	0.5
*Specialist doctor*	21	2.2	0.7	3.0	0.6	2.6	0.5	1.4	0.5	2.7	0.4
*Psychologist*	15	2.2	0.8	2.9	0.7	2.9	0.8	1.7	0.7	2.6	0.5
*Physiotherapist*	27	2.8	0.8	3.3	0.6	3.1	0.6	1.4	0.6	2.9	0.4
*Intern and Resident doctor*	17	3.0	0.9	3.3	0.5	2.6	0.8	1.2	0.5	2.9	0.5
*Reg. healthcare counsellor*	7	2.2	0.4	3.1	0.5	2.9	0.6	1.5	0.4	2.7	0.3
*Rehabilitation coordinator*	1	3.7	---	4.0	---	3.8	---	1.0	---	3.6	---
*Total*:	190	2.8	0.9	3.1	0.6	2.9	0.6	1.5	0.6	2.8	0.5

### Qualitative strand

The final number of participants in the qualitative strand was eight individuals (two males and six females) with varied professional experience and age, comprising two physiotherapists, two registered nurses, one psychologist and three doctors. The analysis process resulted in two categories: Managing EBP in PC and Embracing EBP in PC, based on seven subcategories. A theme labelled “The dilemma of the split between theory and reality” emerged from the concluding abstraction of the manifest result derived from the meaning units, subcategories and categories. The complete theme structure and analysis process are illustrated in Tables 5 and 6.

**Table 5:** Illustration of the analysis process

Meaning unit	Condensation	Code	Subcategory	Category
*“If you want to change something you may have to challenge a senior colleague, not everyone wants to do that.”*	Difficult to change established practices	Difficulty challenging norms	Collegial dialogue	Embracing EBP in PC
*“I believe that there is less micromanagement in PC compared to specialist care. Less guidelines and such.”*	Less micromanagement in PC	Less micromanagement	Reliance on evidence	Embracing EBP in PC
*“Evidence is very much on group level, while we only focus on the individual.”*	Group level evidence instead of individual	Evidence not tailored to the individual	The patient encounter	Managing EBP in PC
*“A strange thing is the opinion that clinical experience is looked upon as the norm when it comes to what works and what doesn’t… It might be generational.”*	Clinical experience most valued	Clinical experience is the norm	Collegial dialogue	Embracing EBP in PC
*“My experience is that we have an increased awareness of the sheer volume of knowledge that you are supposed to possess while simultaneously we have less time to collect this knowledge.”*	More information, less time	More information, less time	Barriers to EBP	Managing EBP in PC

**Table 6:** Qualitative analysis result structure

Subcategory	Category	Theme
*The patient encounter*	Managing EBP in PC	The dilemma of the split between theory and reality
*Evidence vs. Experience*		
*Availability of evidence*		
*Barriers to using EBP*		
*Research climate*	Embracing EBP in PC	
*Collegial dialogue*		
*Reliance on evidence*		

#### Managing EBP in PC

This category comprises four subcategories describing experiences of when and how to use EBP as clinical guidance in everyday clinical practice: The patient encounter, Evidence versus experience, Availability of evidence and Barriers to using EBP.

##### The patient encounter

This subcategory contains statements that indicate how the participants relate to evidence in patient encounters, including gathering, evaluating and conveying evidence-based information to the patient. Other factors mentioned are the difficulty adapting evidence to an individual patient’s unique case and the fact that evidence is presented on group level, thus ignoring each patient’s individuality and unique anamnesis.

*“It often feels as if we don’t give the best treatment because the resources are lacking; instead, we give the least bad treatment. Because there is no room to use the evidence efficiently, even if it does exist.” (Participant 1)*.

*“Evidence is very much at group level, while we only focus on the individual.” (Participant 3)*.

##### Evidence versus experience

Evidence vs. experience covers the issue of when and how to use evidence. Statements concern the question of when it is necessary to take account of more than just evidence. The subcategory describes how to navigate between as well as trusting evidence and experience as the basis for clinical decision-making.

*“I have only been a registered nurse for a year, so I am more inclined to trust the evidence as I have not yet gained much experience.” (participant 8)*.

*“Sometimes experience is a substitute for evidence, but often it´s like a complement.” (Participant 6)*.

##### Availability of evidence

The main content of this subcategory is the ease of finding and accessing evidence. The availability of evidence seemed to vary in PC depending on the nature of the diagnosis. In general, the participants considered that relevant evidence was hard to find or interpret. The preferred form of evidence was a review of original research articles. Evidence-based internal guidelines were requested for a greater number of areas, as well as more rapid updating of existing guidelines when new evidence emerged.

*“I would like to say that it depends… The availability of evidence differs depending on speciality. Both in terms of accessibility and trustworthiness.” (Participant 5)*.

*“I frequently read guidelines and PMs, less often research articles, as their content reaches us in summarised form after review by the county.” (Participant 7)*.

##### Barriers to using EBP

This subcategory contains the main barriers to using EBP as described by the participants. The most frequently mentioned barrier was lack of time, i.e., time with patients and for keeping updated on research to gain a better understanding of the situation. The underlying reason for lack of time seems to be the perceived pressure to keep up with the volume of work.

*“You are supposed to have the time to thoroughly read and evaluate new evidence and whether the methods are sound, but that time is not included in the clinical reality.” (Participant 4)*.

*“My experience is that we have an increased awareness of the sheer volume of knowledge that you are supposed to possess, while simultaneously having less time to collect it.” (Participant 2)*.

##### Embracing EBP in PC

The category “Embracing EBP in PC” comprises three subcategories: Research climate, Collegial dialogue and Reliance on evidence. The similarity between these subcategories is that they describe the opportunities to embrace the concept and practice of EBP in the clinical setting by outlining the research climate in PC in general, the collegial dialogue about evidence and new knowledge, and finally, how to know when to rely on evidence in daily practice.

##### Research climate

This subcategory comprises statements outlining attitudes towards research per se as well as conducting and utilising it. The general attitude in relation to research was that it is necessary, especially in PC due to its large size. Attitudes pertaining to the impact of research in daily clinical practice are also described.

*“People don’t see the possibility. There are few academic supervisors available, and you are not offered the opportunity to conduct research. If you want to, you must fight pretty hard for it.” (Participant 1)*.

*“Primary care needs more research but it [the management] does not understand that conducting research must be rewarding.” (participant 2)*.

##### Collegial dialogue

This subcategory encompasses the perceptions of being able to discuss evidence in the PC centre as experienced and expressed by the participants and their colleagues. The general impression is one of interest in discussing evidence amongst colleagues but lacking real opportunities to do so. Some also stated that collegial dialogue is difficult to achieve due to a few colleagues’ lack of interest in discussing therapies and evidence.

*“Of course, you discuss it [evidence], but it is hard to discuss the quality of the articles.” (Participant 1)*.

*“If you want to change something, you may have to challenge a senior colleague; not everyone wants to do that.” (Participant 4)*.

##### Reliance on evidence

The subcategory contains our participants’ statements on the value of evidence and their trust in it. Evidence is generally seen as something that represents knowledge, security and the gold standard for clinical decisions in everyday practice. Nevertheless, some statements diverged from the commonly expressed consensus, with participants stating that evidence decreases in value when one’s professional experience increases.

*“In education it is stressed that everything should be evidence-based: you should be able to build your choice of theory and methods on the basis of evidence. But how this is fulfilled in practice seems to be a completely different story.” (Participant 4)*.

*“I believe that there is less micromanagement in primary care compared to specialist care. Less guidelines and such.” (Participant 6)*.

### Overarching theme: The dilemma of the split between theory and reality

The overarching theme was formulated out of interview data formed into categories, subcategories and codes, all following a “red thread” connecting the manifest text to the analysed latent meaning. The theme of being torn between two realities that seems hard to overbridge. In the interviews, the participants stated that they had knowledge of EBP and generally expressed a positive attitude towards it, adding that they knew they were supposed to base every decision on evidence. However, barriers in the form of a lack of resources, perceived disinterest from colleagues in discussing and sometimes implementing evidence and tardiness of information dissemination in the organisation made compliance with and implementation of EBP seem like an extra task on top of an already heavy workload.

### Convergent result

**Fig. 1:**
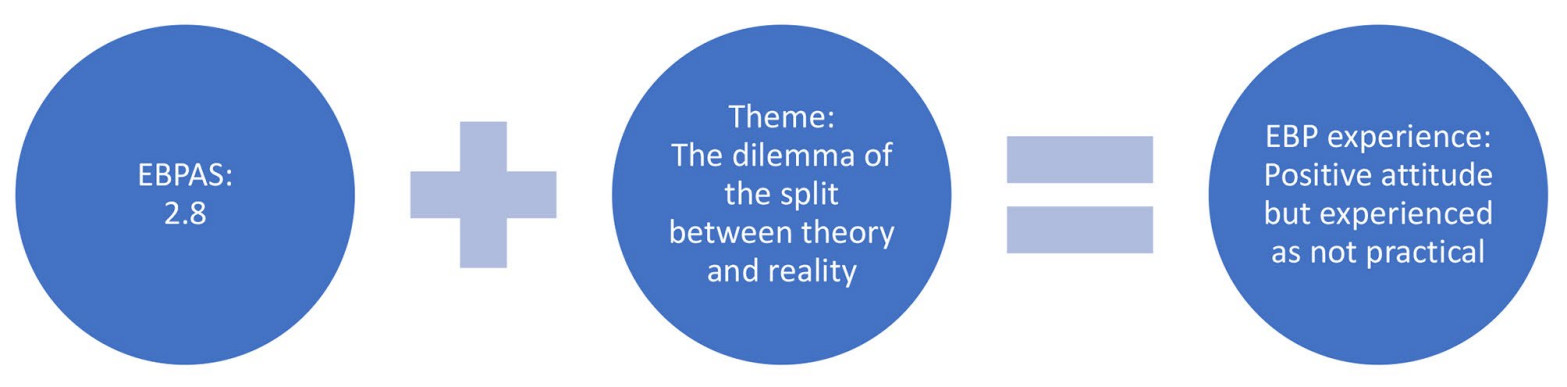
Illustration of the convergent result

On an item level, the content of each EBPAS subdomain was found to correspond with one or more subcategories in the qualitative content analysis, as illustrated by the conjunction table below (Table 7). The EBPAS is designed to quantitatively describe attitudes towards EBP that could be translated into qualitative terms in the form of a single mean value. In turn, the overarching theme and underlying categories and subcategories lend themselves to interpretation based on the defined EBPAS subdomain. When combined, these two findings form new aspects of the results. Figure 1 illustrates the convergent result and its inherent parts.

**Table 7:** The content of EBAS domains in each qualitative subcategory

Qualitative subcategory	Requirements	Appeal	Openness	Divergence
*The patient encounter and EBP*	X		X	X
*Evidence versus experience*	X		X	X
*Availability of evidence*	X	X		X
*Barriers to using EBP*	X	X	X	X
*Research climate*				X
*Collegial dialogue*		X	X	X
*Reliance on evidence*		X	X	X

## Discussion

This mixed methods study revealed that HPs’ attitudes to EBP in PC were generally positive, which is in line with the results of earlier research [3, 5, 7, 20, 24, 25]. However, it is important to bear the self-reported nature of the qualitative data in mind. The positive attitude might be a “politically correct” answer due to the fact that EBP is firmly anchored in modern medicine and legislation [15, 21]. In view of this, there ought to be a conformity bias that influences the respondents. For example, in a study performed in 2021, 68% stated the obligation to follow guidelines, while at the same time exhibiting a neutral or low level of enthusiasm for EBP [21]. The same influence of legislation or policies can be seen in Swedish primary care, as our earlier research of EBP knowledge amongst primary care managers shows [8].

When practising EBP, there might also be a problem regarding familiarity with the EBP concept and the ability to understand crucial terminology [3, 5]. The degree of understanding of concepts could be seen as being either a result of educational or clinical exposure or professional experience [5]. This is supported by the quantitative results (Table 3); experience leads to the ability to choose when to practise EBP.

The results from our study and the literature [3] reveal a high level of self-reported familiarity with EBP and its concepts that is failing when implemented into clinical action [3]. Moreover, the same review, as well as several others, also indicates some confusion about and disparities in the definition of EBP and its concepts [3, 5, 6, 20]. Our participants stated that EBP is primarily used to justify clinical decisions. When asked what evidence was used, most of the participants referred to scientific articles. This contradiction can be seen as evidence of a belief in using EBP. Although merely supporting clinical decisions based on guidelines or article reviews. Consequently, it can contribute to upholding clinical traditions and customs of what in essence is an opinion-based practice [3]. It is our interpretation that divergence from EBP may be the ultimate outcome of a lack of education and clinical experience, resulting in a more pragmatic way of practising EBP with the help of colleagues, guidelines, policies etc. In this way, the efficiency and reliability of EBP and its concepts may be impaired and made even worse by the misconceptions of EBP and how to integrate it in clinical practice, thus contributing to an ineffective and potential degradation of care quality [3].

Most frequently, the lack of time to evaluate the information pertaining to each patient was stated to be of increasing importance both in the literature and in our interviews [3, 5, 7, 8, 20, 25, 27]. In addition, the lack of time to practise EBP in an appropriate manner was repeatedly mentioned and has been shown to affect PHC management [7]. Worth mentioning in this context is the connection between managerial ethical stress that occurs when the knowledge of the impact of lacking resources clashes with the wish to allow co-workers enough time, on the one hand, and financial resources to be able to fully practise EBP, on the other [7]. This lack of time and economic resources to be able to practise EBP could be seen as being passed on from management to all co-workers. The time resource may thus be, at least perceived, to be set and beyond the control of most co-workers [7], thus contributing to the lack of opportunities to practise EBP. In the long term, this will influence the quality of care in PC [3, 4, 7].

A scarcity of mainly non-existent information resources in the form of databases and applications was also mentioned by HPs as a hindrance to the practise of EBP [5, 21]. The specific lack of informational resources was not mentioned by the participants, although the lack of knowledge about where to find evidence might be a result of this. It would appear that PC has a supply problem regarding evidence adapted to the PC setting with comorbidities and the complex context that exists within PC [30]. This was also an opinion voiced by our participants. Apart from the perceived lack of research, the desire for evidence could depend on several other factors, such individual research skills, lack of technology and education [20, 21, 30].

Focusing upon the quantitative strand, some interesting associations were found. For instance, the variables “education” and “experience” were comparable with the EBPAS subdomain of divergence (Table 3). This could indicate that HPs with lower education seem more likely to deviate from evidence (Table 3). A similar association could be seen in terms of divergence and education, yielding an increased divergence score with growing professional experience. For example, we can see that assistant nurses, a group with relatively low educational requirements, have a higher total EBPAS score than most other occupations. This is interesting as most of these participants had less than five years of education. Thus, both the lowest and one of the most highly educated professions had the highest total EBPAS scores, but educational value could be traced when scrutinising the divergence subdomain. The attitude towards practising EBP can thus be seen as influenced by at least two factors: exposure and education. The high EBPAS score of our assistant nurses could be the result of the same phenomenon described by a study in 2021 [21]. That is, occupational experience provides more exposure to EBP and EBP-related thinking, in turn influencing the attitude towards it [21]. In 2004 Aarons et al. mentioned education as an influential factor for how HPs perceive EBP [31]. More education could mean that an individual HP considers EBP less burdensome compared to colleagues with a lower educational level [31].

The attitude towards research and EBP among managerial staff has been found to be of great importance [7]. A positive attitude towards research and its utilisation should permeate from the managerial down to the bottom level of the hierarchy [25]. In this way, managers can influence the local research culture, which in turn creates an incentive to use and conduct research [7, 21, 25].

Participants in the present study described a research climate that is perceived as tough for creating the opportunity to conduct research or keep abreast of published research. This is also an acknowledged problem amongst HPs internationally [5, 21, 37]. The unwillingness to risk confrontation with colleagues when trying to discuss EBP or suggest a change of practice seemed daunting enough to prevent the participants from discussing the topic with colleagues. The social climate is ultimately a result of the management of the PHC, which has been shown in earlier studies [7–10].

The split between theory and reality was also supported by the EBPAS scores, especially the presence of divergence in all categories identified by the content analysis. In line with the HPs’ statements, divergence is the subdomain that most clearly relates to their tendency to adhere or not adhere to evidence. Our results showed less divergence among participants with a higher education. However, the divergence seemed to increase in line with experience and the number of years in the profession (Table 3). The comparison between our two groups of doctors (interns and specialists) supports the finding that increased experience results in more autonomy in relation to evidence and guidelines (Table 4). Thus, there is a conflict between following guidelines that may be outdated or unavailable and following one’s own professional experience and established anecdotal therapy methods. In order to advance the understanding and acceptance of EBP in PC at all levels, there seems to be a great need for collegial and organisational dialogue about evidence and its use. According to the participants in our study, this need appears to be largely unmet.

Previous studies have reported resistance towards the implementation of EBP due to a lack of individual motivation, organisational support and external motivation [5, 20, 21, 25, 30]. It is safe to say that our study confirms this description and underlines the need for further research relating to the role of individual HPs in providing quality care despite the organizational climate. Such research can even be considered vital for ensuring high-quality care in the future.

### Strengths and weaknesses of the study

The convergent mixed methods design was chosen due to being the most appropriate way to describe our results, bearing in mind that the data collection in both strands was performed separately and sequentially. The pragmatic triangulation enabled by this method is optimal for describing and merging the qualitative and quantitative research paradigms employed.

The original EBPAS (EBPAS-15) was chosen for the quantitative strand. The EBPAS-50 and EBPAS-36 are both based on the EBPAS-15. The reason for the choice of the original tool was that it has good psychometric coverage, is validated and furthermore, is the only one that has been subjected to confirmative factor analysis in a Swedish setting [32]. The EBPAS 36 is a reduced version of the EBPAS 50 scale used in Norwegian and US settings [26].

The conclusion from earlier research employing the EBPAS is that while there are less robust results in the Divergence subscale [32], the total psychometric reliance is good. To remedy this lack of utility in the subdomains, the present study was designed as a convergent mixed methods study in order to strengthen the EBPAS analysis by the addition of interviews analysed by means of qualitative content analysis. This contributes to increased knowledge of divergence because its presence in all qualitative subcategories could be interpreted as confirming the validity of the EBPAS subdomain (Table 7; Fig. 1).

In qualitative research, trustworthiness is measured in different ways. Graneheim and Lundman present trustworthiness as a compound of the following concepts [34]: Credibility (focus of the research and how well data and analysis meet the aim), Dependability (degree of data change over time and influence of researcher bias during the analysis process) and finally, transferability (how well the findings can be transferred to other groups and populations) [34]. In our study these factors have been included in the study design, for instance in choosing participants, describing the qualitative analysis process as clearly as possible and finally discussing the strengths and weaknesses of the study.

The unequal conditions for the qualitative interviews are not ideal. Although efforts were made to recruit two focus groups, there was simply no way to arrange for the participants to meet at the same location. As there was no managerial support, participants had to take part in our study in their free time. This underlines the fact that participation in research is considered extracurricular and something to be done in one’s own spare time.

The qualitative content analysis moves between the manifest and the latent content. This means that the process of turning verbatim text into increasingly abstract codes, categories and a final theme is always subject to the researchers’ interpretation [35]. The interpretative nature of this process implies that the method could result in different outcomes depending on the perspectives of the researchers performing the analysis. This might be visible in the abstraction process and the overarching theme and is also the reason a thorough description of the research team and the process is provided.

The strengths of the study rest upon the merged results of a validated instrument (EBPAS) and in-depth interviews with HPs. The convergent mixed method analysis of quantitative EBPAS results and qualitative interview data helps to deepen the understanding of the complexity of evidence-based medicine in primary care praxis.

This combination of quantitative psychometric measures coupled with results from the qualitative content analysis provides a fuller image of HPs’ experiences of working in accordance with EBP. In addition, the results shed light on the complicated process of managing information and implementing the EBP process in a complex organisation.

One weakness of the study might be the somewhat small sample size. However, this may not necessarily be a hindrance due to the willingness of each participant to share information and the rich content of the interviews. Even a small sample might be deemed adequate if selected with care and the data are considered sufficiently valid. Although the participants’ responses revealed a degree of saturation, the inclusion of more participants would probably have strengthened the reliability of the study. However, interest in participation was low, as the formal invitation to participate was sent to the whole population of 625 individuals and only 190 expressed an interest. This might also be a result of lack of time, as HPs may feel the need to prioritise patients rather than research, which once again highlights the frequently mentioned time constraints.

## Conclusions

Successful implementation of EBP is dependent on common definitions and practices on individual, organisational and external levels. Some degree of education, be it theoretical or practical, is necessary to be able to practise and discuss EBP. A positive research climate enhances collegial debate about EBP as well as its effective use and growth. Ineffective use of EBP due to barriers in either of these factors is a severe hindrance to its growth. This in turn poses a serious threat to the quality of care and, ultimately, patient outcomes.

### Electronic supplementary material

Below is the link to the electronic supplementary material.

Appendix 1: EBPAS-Subscales, items and plain text question

Appendix 2: Interview guide

## Data Availability

The datasets used and analysed during the present study are available from the corresponding author on reasonable request.

## List of Abbreviations

EBP: Evidence–Based Practice
EBPAS: Evidence–Based Practice Attitude Scale
HP: Healthcare professional
PC: Primary healthcare
SD: Standard deviation
