# Supplementary material for: The dilemma of the split between theory and reality as experienced by primary healthcare professionals: a mixed methods study of evidence-based practice in a primary care context
Source: BMC Prim Care. 2024 Jan 5;25:13. doi: 10.1186/s12875-023-02237-9 (PMC10768255; doi:10.1186/s12875-023-02237-9)
Supplement: Supplementary file 1 — Appendix 1: EBPAS-Subscales, items and plain text question [file 12875_2023_2237_MOESM1_ESM.docx]

| Appendix 1 – EBPAS-Subscales, items and plain text question. | | |
| --- | --- | --- |
| **Subscale** | **Item** | **Question:** |
| *Requirements* | 12 | it was required by your agency? |
|  | 11 | it was required by your supervisor? |
|  | 13 | it was required by your state? |
| *Appeal* | 14 | it was being used by colleagues who were happy with it? |
|  | 15 | you felt you had enough training to use it correctly? |
|  | 9 | it was intuitively appealing? |
|  | 10 | it “made sense” to you? |
| *Openness* | 1 | I like to try new types of therapy/interventions in order to better help my clients. |
|  | 2 | I am willing to try new types of therapy/interventions even if I have to follow a treatment manual. |
|  | 8 | I would try a new therapy/intervention even if it were very different from what I am used to doing |
|  | 4 | I am willing to use new and different types of therapy/interventions developed by researchers. |
| *Divergence* | 5 | Research based treatments/interventions are not clinically useful. |
|  | 6 | Clinical experience is more important than using manualized therapy/interventions. |
|  | 7 | I would not use manualized therapy/interventions. |
|  | 3 | I know better than academic researchers how to care for my clients. |
