# Supplementary material for: The dilemma of the split between theory and reality as experienced by primary healthcare professionals: a mixed methods study of evidence-based practice in a primary care context
Source: BMC Prim Care. 2024 Jan 5;25:13. doi: 10.1186/s12875-023-02237-9 (PMC10768255; doi:10.1186/s12875-023-02237-9)
Supplement: Supplementary file 2 — Appendix 2: Interview guide [file 12875_2023_2237_MOESM2_ESM.docx]

Appendix 2 – Interview guide

**Evidence and EBP**

- What is your definition of evidence and evidence-based practice?

Follow up:

- - Relevance
  - Trust and availability.
  - Application
  - Primary care
- Could you tell me about the general discussion climate amongst colleagues regarding;
  - Relevance of evidence.
  - Trust and availability of evidence.
  - Primary care and evidence.

**Research**

- Could you describe the general view amongst colleagues on research and using research-based evidence at your PHC?

Follow up:

- - Evidence
  - EBP
- Could you tell me if you have experienced any change in this view?

Follow up:

- - Evidence
  - EBP

**Support**

- Could you tell me about the general discussion climate with organizational management regarding;
  - Relevance of evidence.
  - Evidence based practice and primary care.
- How do you perceive the support from management when it comes to working according to evidence-based practice?

Follow up:

- - Managerial
  - Organizational

**Patient meeting**

- Could you describe how evidence relates to a typical patient meeting?
- In what way do you use evidence in these situations?

Follow up:

- - EBP
